# Effects of Co-Solvent Nature and Acid Concentration in the Size and Morphology of Wrinkled Mesoporous Silica Nanoparticles for Drug Delivery Applications

**DOI:** 10.3390/molecules26144186

**Published:** 2021-07-09

**Authors:** Jessica Andrea Flood-Garibay, Miguel A. Méndez-Rojas

**Affiliations:** Department of Chemical & Biological Sciences and Laboratory of Nanotechnology and Molecular Biomedicine Research, School of Sciences, Universidad de las Américas Puebla, San Andrés Cholula, Puebla 72810, Mexico; jessicaa.floodgy@udlap.mx

**Keywords:** wrinkled mesoporous silica (WMS), co-solvent, drug delivery

## Abstract

Hierarchically porous materials, such as wrinkled mesoporous silica (WMS), have gained interest in the last couple of decades, because of their wide range of applications in fields such as nanomedicine, energy, and catalysis. The mechanism of formation of these nanostructures is not fully understood, despite various groups reporting very comprehensive studies. Furthermore, achieving particle diameters of 100 nm or less has proven difficult. In this study, the effects on particle size, pore size, and particle morphology of several co-solvents were evaluated. Additionally, varying concentrations of acid during synthesis affected the particle sizes, yielding particles smaller than 100 nm. The morphology and physical properties of the nanoparticles were characterized by X-ray diffraction (XRD), Fourier transform infrared spectroscopy (FTIR), scanning electron microscopy (SEM), and dynamic light scattering (DLS). Homogeneous and spherical WMS, with the desired radial wrinkle morphology and particle sizes smaller than 100 nm, were obtained. The effect of the nature of the co-solvents and the concentration of acid are explained within the frame of previously reported mechanisms of formation, to further elucidate this intricate process.

## 1. Introduction

Over the past three decades, there has been significant progress in the synthesis, design, and use of hierarchically porous materials, in areas such as nanomedicine (e.g., drug delivery, bioimaging, photothermal ablation, protein, and gene delivery, etc.) [1]; immobilization of large molecules, such as proteins [2]; catalysis [3]; solar-energy harvesting (e.g., photocatalysis, solar cells, etc.) [4]; energy storage; CO_2_ capture, as smart nanocarriers of corrosion inhibitors [5]; and other industrial applications. Hierarchically porous materials are made of interconnected pores with different lengths and diameters (ranging from micro (<2 nm), meso (2–50 nm) to macropores (>50 nm)) that present a multimodal hierarchically porous structure. These types of material have advantageous features, such as numerous synthetic approaches, tunable porous structures, controllable macroscopic morphologies, multiple functions, and many potential uses [6]. Regarding nanomedicine, nanoparticle-based delivery systems are promising targeted drug delivery systems (DDS), which have recently earned great attention for the selective delivery of therapeutic agents [7]. Currently, the delivery systems that are available for clinical use are mainly organic materials, such as liposomes, other lipid formulations, and polymers [8,9,10,11]. Nonetheless, their applications for drug and macromolecule delivery are hindered by their intrinsic instability and limited drug-loading capacity [12,13].

Mesoporous silica nanoparticles (MSN) have a large surface area (over 700 m^2^/g), tunable pore volume, and sizes that can be controlled by varying the nature of the surfactant molecule, the reagents stoichiometry, the auxiliary chemicals, the reaction conditions, or even by post-synthesis functionalization techniques [6,14,15]. The control of the MSN particle diameter is of great significance, as it may affect cell uptake, cytotoxicity, and dispersibility [16,17,18,19]. Pore size control is important because it affects both the confinement effect of the pores and the accessibility of incoming guest molecules [20,21,22]. Furthermore, the silanol (Si–OH) residues on the MSN surface facilitate chemical modification, which can be used to covalently bind drug molecules or functionalize the surface with antibodies, aptamers, small molecules, stimulus-sensitive materials, and luminescent or fluorescent materials, which can lead to intelligent and multifunctional properties [15,23]. Additionally, compared to niosomes, liposomes, and dendrimers, MSNs are more stable against external factors, such as thermal- or pH-dependent degradation and mechanical stress, due to their strong Si–O bond [24].

Many synthetic procedures are available for the preparation of mesoporous materials, which can be classified as soft-templating, hard-templating, or template-free approaches [25,26,27]. Surfactants serve as a template throughout the nanocasting process by forming regular super-structured micelles, which feature structural motifs on the nanometer scale [28]. This method requires that a precursor with similar polarity condenses around the micelles to replace the solvent, after which all the templates (surfactant molecules) are removed. This approach has been used to synthesize porous metals and mesoporous silica [29], as well as MCM-40 and SBA-15 [30].

Regarding the particle size control of non-mesoporous silica nanoparticles, the task is usually achieved by controlling the addition rate of the silicon source [31]. The control of the hydrolysis rate of the silicon precursor is the most important factor, and it can be achieved by varying the concentration of the catalyst, the pH of the reaction, adding alcohols as co-solvents, or by varying the nature of the alkoxysilanes used [32,33,34]. The particle size becomes larger when the rates of hydrolysis of alkoxysilanes are slowed [35]. The variation in the structure and morphology of the products are also affected by the condensation rates, which can be controlled by the introduction of hydrophobicity into the reaction media, with the addition of different types of alcohols to the reaction as co-solvents [36,37].

In recent years, wrinkled mesoporous silica (WMS) has attracted interest due to its similarities with MSN, plus its unique characteristics, such as a fibrous surface morphology that may be physiologically relevant [34]. WMS exhibits good thermal stability (from 400 to 950 °C), great hydrothermal stability, and high mechanical stability, even after mechanical compression up to a pressure of 216 MPa [38]. Compared to compact, small-ordered, and monomodal mesopores in traditional MCM and SBA mesoporous silica spheres, WMS feature hierarchical open-pore structures with a unique radial and wrinkled conical shape that provides highly accessible sites with routes for adjustable mass transport that allow better reagent diffusion and higher drug loads when used for drug administration [39]. It was recently demonstrated that magnetic nanoparticles could be embedded into the mesoporous structure, which can act as multifunctional drug carriers and medical imaging agents [40]. However, it has proven difficult to prepare WMS with large pores and a well-defined spherical shape below 100 nm in diameter; this is a threshold size that is advantageous for both biomedical and catalytic applications [41].

There are several strategies for WMS synthesis (microemulsion [42], microwave-assisted hydrothermal [38], and the self-assembly [43] methods). In the microemulsion method, the ternary systems show four types of phase behavior (the so-called “Winsor System”), determined by the surfactant concentration and the Winsor R-value [43]. According to Moon’s work, the interparticle connective structures and internal morphologies can be adjusted using the different ternary systems as structure-directing templates. When the system is surfactant-rich, a single-phase microemulsion is formed (Winsor type IV), and MSN with no radial wrinkles are formed. It was observed that independent mesopores of silica nanospheres began to progressively interconnect and convert into wrinkled structures as the amount of oil increased in the system (Winsor II and III). The type-III system consists of a bicontinuous microemulsion (with an additional water layer, and an oil layer) where the nanospheres are still well-formed and the inter-wrinkle distance tends to be the most sizable [42,44]. However, although Moon’s work explains the changes in morphology and pore size, the proposed mechanism does not answer some key questions, such as the variation in the sphere size, dependent on the reaction conditions, and the dendritic nature of the silica fibers.

Similarly, Gustaffson et al. proposed a very similar mechanism of formation, in which each oil drop in the O/W emulsion formed is a water-in-oil microemulsion. Their model explains that the surfactant is present both around the oil drops and around the microemulsion droplets present within the oil drops, while the silica precursor (TEOS) is contained in the oil phase. In this system, most of the interface is within the oil drops, as the oil–water interface in such a system is very large. As in the previous model, when TEOS is exposed to water, the precursor will hydrolyze at all the available oil–water interfaces, which will lead to a gradual transition of the droplet structure within the diminishing oil drops, into a structure composed of small, elongated water channels formed by the self-assembly of the surfactant. The authors continue to explain that these structures will gradually condense to form long, narrow silica threads, protruding through what remains of the oil drops when all the TEOS has been consumed, forming a three-dimensional silica network. It is easy to notice the overlap between Moon’s and Gustaffson’s mechanism, in a point known as the transition from a water-in-oil microemulsion into a bicontinuous microemulsion [41].

The clear understanding of the intricate interplay between the solvent, surfactant, co-surfactant, base, and other reaction parameters that control the mechanism of formation of WMS remains an unsolved challenge, although a few works have systematically investigated it. Further work on the elucidation of the consequences of changes to the reaction conditions and their effects on the nanoparticle size, morphology, pore size distribution, and inter-pore distance can potentially aid in the unambiguous and fundamental understanding of the mechanism of formation. In this study, we prepare several WMS spheres by adding different types of organic molecules, as co-solvents, to the biphasic suspension formed by cetylpyridinium bromide (CPB), tetraethyl orthosilicate (TEOS), a base, and cyclohexane, to determine their effect on nanoparticle size, pore size, and general morphology. It was found that the smallest WMS sizes were achieved when adding isopropanol and ethylene glycol (a co-solvent that had not been previously used before for these types of reactions), while octanol, ethanol, and glycerol yielded particle sizes above 150 nm. The reactions with isopropanol and ethylene glycol were further studied, with the addition of varying amounts of HCl, which is a strategy that produced WMS with diameters well below 100 nm.

## 2. Results

**Figure 1:**
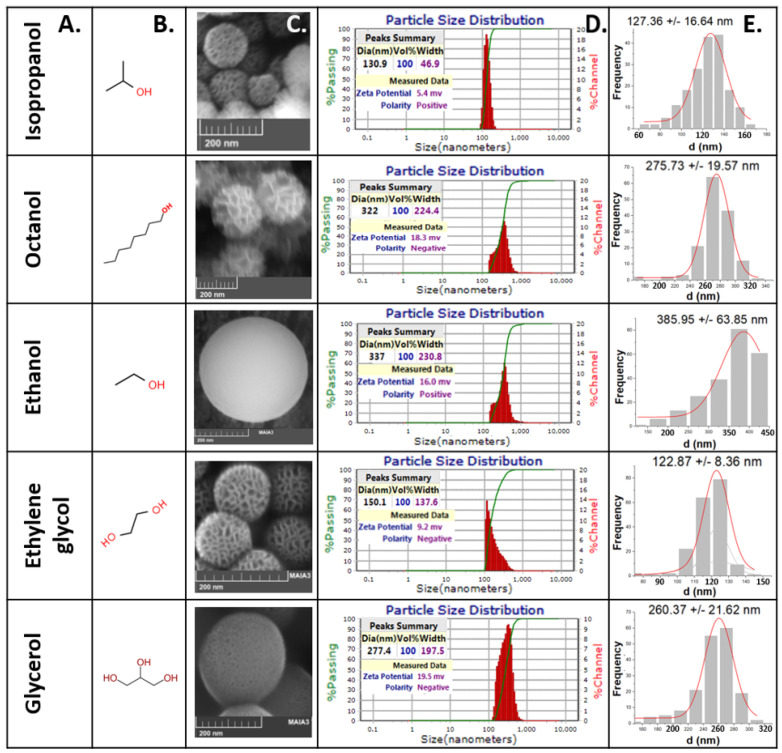
WMS typical homogeneity and size dispersion for different synthesis done with varying co-solvents. (**A**) Co-solvent, (**B**) structure of co-solvent, (**C**) SEM micrographs of WMS nanospheres, (**D**) size dispersion, hydrodynamic radius and Z potential acquired by DLS, and (**E**) scheme 100 nanoparticles from SEM micrographs using ImageJ.

The size and morphology of the nanoparticles were analyzed using SEM for all syntheses, to ensure reproducibility of the process. In all the cases, the obtained solid systems are spherical and monodispersed; nevertheless, the observed wrinkled patterned surface changes depending on the co-solvent used in the synthesis. Radial wrinkles are visible when using isopropanol, octanol, and ethylene glycol during the synthesis. By inspecting the SEM micrographs of these WMS spheres, pores are visually evident, with sizes in the 5 to 20 nm range when isopropanol and ethylene glycol are used, and between 20 to 100 nm when using octanol. WMS synthesized in the presence of ethanol and glycerol seem to have a less fibrous superficial morphology, with a smother solid surface presenting very small micropores that were impossible to measure precisely with the image processing software (ImageJ). When only the co-solvent was varied (Figure 1), the size of the NPs was affected; octanol, ethanol, and glycerol rendered sizes between 250 and 350 nm. In contrast, when isopropanol and ethylene glycol were used as co-solvents, nanospheres with sizes around 120–130 nm were obtained. As determined from the DLS hydrodynamic diameter measurements, none of the WMS nanospheres present agglomeration, which is independent of the co-solvent used in the synthesis. In all the cases, the zeta potential values (ζ) were low (5–19 mV).

**Figure 2:**
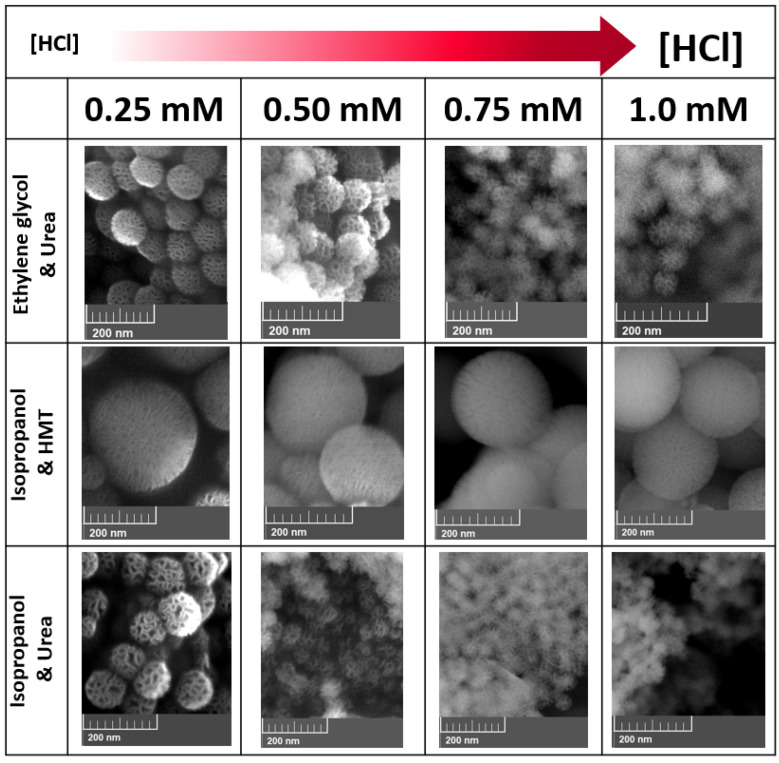
WMS typical homogeneity and size dispersion as seen by SEM for different synthesis performed using varying co-solvents and bases in the presence of distinct concentrations of HCl.

When varying the amounts of HCl that were added to the reaction mixture (Figure 2), the series using isopropanol and HMT resulted in NPs with sizes between 220 and 270 nm. For these series, the sizes were homogeneous and the NPs were spherical, with a radial wrinkled morphology that created pores that measured between 5 and 15 nm (as determined by ImageJ analysis). The series of reactions using ethylene glycol and urea, as well as those using isopropanol and urea, resulted in NPs with sizes between 100 and 40 nm. In both the reaction series (isopropanol/urea, and ethylene glycol/urea), the NP sizes decreased when large concentrations of acid were added.

**Figure 3:**
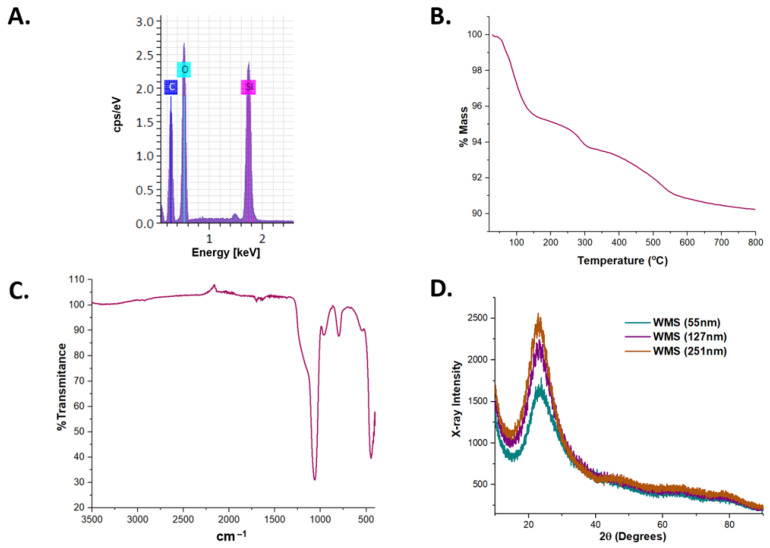
Typical characterization of WMS systems. (**A**) Chemical composition by EDS. (**B**) Thermal analysis by TGA in inert conditions. (**C**) FT-IR spectra. (**D**) Wide-angle XRD pattern.

WMS were characterized before and after heating. The WMS particles that were heated at 800 °C under an inert N_2_ atmosphere were examined under SEM. Comparison among the images did not show any changes in morphology, shape, or size, indicating that the WMS are thermally stable. The EDS analysis showed the expected chemical composition for the WMS (O and Si). The C and Al signals were due to the mounting substrate (adhesive carbon tape on an aluminum pin) (Figure 3A). The FT-IR spectra (Figure 3C) of both the washed and dried products only show vibrations corresponding to the silane groups at ν(O–Si–O) 1060 cm^−1^ and ν(Si–OH) 956 cm^−1^. TGA allows the identification of thermal-related changes in the sample (dehydration, decomposition of the physically and chemically absorbed molecules, loss of volatile fragments, phase transitions, among others). TGA of the WMS (Figure 3B) shows a continuous weight loss for all the samples in the temperature range of 20–120 °C, which is attributed to dehydration of the samples. Weight loss in the 270–310 °C range can be associated with thermally protected remnants of the CPB surfactant trapped inside the mesoporous structure. Pure CPB decomposes rapidly in the range from 200 to 255 °C (see Figure S1 in the Supplementary Materials for a TGA curve corresponding to pure CPB). The weight loss occurring in the 300–500 °C range has been attributed to the loss of hydrogen-bonded and isolated hydroxyl (OH) groups present on the mesoporous silica surface [45,46].

The WAXRD showed normal occasional fluctuations of the electronic density, because of the long-range ordering of the pores in the material, revealed by a strong diffraction peak in the range of 17–30° 2θ (Figure 3D) [47,48]. It is important to mention that the powder DRX patterns for WMS with different sizes were obtained, all of which had the same peak in 2θ, which only varies in intensity between the different samples.

**Figure 4:**
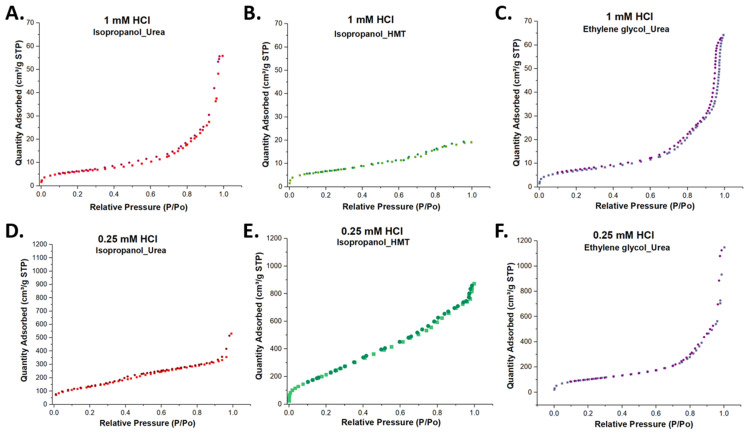
N_2_ adsorption–desorption isotherm for WMS systems synthesized with different HCl concentrations. The systems synthesized with 1 mM HCl, and (**A**) isopropanol as a co-solvent and urea as a base, (**B**) isopropanol as a co-solvent and HMT as a base, and (**C**) ethylene glycol as a co-solvent and urea as a base. The systems synthesized with 0.25 mM HCl, and (**D**) isopropanol as a co-solvent and urea as a base, (**E**) isopropanol as a co-solvent and HMT as a base, and (**F**) ethylene glycol as a co-solvent and urea as a base.

The adsorption and desorption of N_2_ were used to determine the surface area of the WMS based on the BET analysis of the adsorption isotherms. This technique has been widely used for non-uniform mesoporous silica materials, for their textural (surface area and porosity) characterization [49]. N_2_ adsorption–desorption isotherms for the series of WMS synthesized using different co-solvents and bases were recorded (Figure 4). For the isopropanol/HMT synthesis, the hysteresis loop exhibited is type II and starts at high pressures P/P_0_ = 0.7, with a blind-hole type of pore indicated by the closeness of the adsorption and desorption trace. In the case of the isopropanol/urea and ethylene glycol/urea synthesis, the hysteresis loops are type IV, starting about P/P_0_ = 0.4, and they are spread over a wide range, which is consistent with the wide pore size distribution that can be also seen in Table 1.

Isotherms with similar hysteresis have been reported for other mesoporous silica nanomaterials, such as the MCM-41 type with 2 nm and 3 nm pore sizes, SBA with an 8 nm pore size, and WMS with similar pore sizes [36,50]. After the BET analysis of the N_2_ adsorption isotherm, the typical surface area for these systems was determined to be in a range between 287 and 915 m^2^/g; this is typical for these types of mesoporous nanoparticles [50,51].

## 3. Discussion

WMS were synthesized following the microemulsion method reported by Moon [42] and Munaweera [52]. The method makes use of a Winsor ternary system type III that forms a bicontinuous microemulsion from which superfluous oil and water separate. Within the dispersed aqueous phase of the emulsion, the silica-forming reaction began with the hydrolysis of TEOS, which is controlled by the concentration and nature of the basic species (HMT, urea). Within the continuous oil phase, the CPB micelles form and auto assemble into what will become the pores of the nanostructure. The interconnected pores generate the wrinkled structure; the inter-wrinkle distance is determined by the co-solvent added to the reaction [52,53]. In the second step, the obtained WMS nanospheres are reacted with HCl in ethanol to wash off the organic CPB template, leaving behind the corresponding hollow pores.

**Figure 5:**
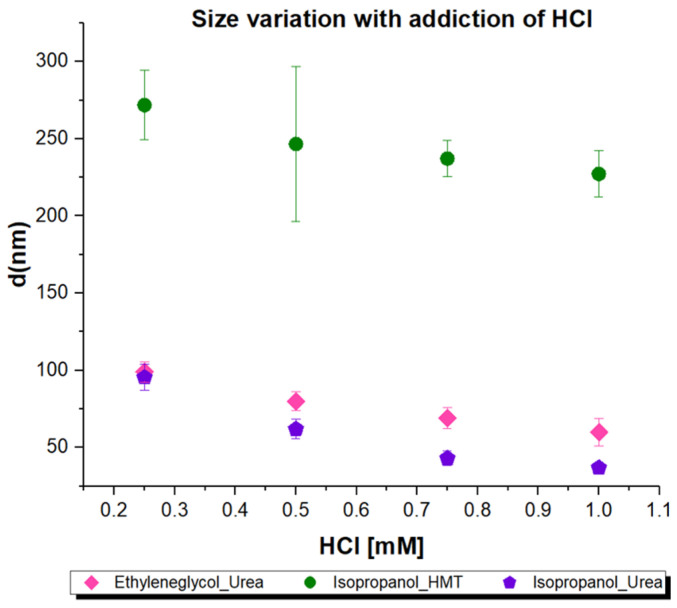
Diameter variation dependent on concentration of HCl added to the three series of synthesis.

To tune the synthetic conditions to produce WMS with sizes smaller than 100 nm, different co-solvents were tried, as well as the simultaneous addition of various concentrations of HCl at the beginning of some of the reactions. As a first step, the nature of the co-solvent and base was varied, to evaluate if they induced any changes in the size and morphology of the WMS. First, reactions were performed using equimolar amounts of ethanol, octanol, isopropanol, ethylene glycol, and glycerol, to obtain different products under the same reaction conditions. As examined below, it was found that the smaller sizes of NPs were achieved when using isopropanol and ethylene glycol. When octanol, ethanol, and glycerol were used, particle sizes were above the desired threshold. As none of the NPs were still under 100 nm, the systems containing isopropanol and ethylene glycol were studied, now adding different amounts of HCl to accelerate the silanol condensation reaction, promoting the initial nucleation reaction and hopefully forming hard shells around the initial smaller microemulsion droplets that would give rise to smaller NPs. As previously discussed, this strategy produced WMS well below 100 nm (Figure 5). This last series of reactions was also evaluated using an alternate base, HMT. This compound was used because, apart from being a Lewis base whose basicity depends on the pH of the solvent, it is soluble in both aqueous mixtures and polar organic solvents. This compound has previously been used as a pore size modifier in carbon cryogels, affecting porosity [54]. As a weak base, HMT slows down the hydrolysis reaction, facilitating the growth of silica nanoparticles around the self-assembled template generated by the CPB surfactant, and generating larger particles [35].

The reactions run using the co-solvents glycerol, ethanol, and octanol yield mesoporous nanospheres with similar sizes, but different surface morphologies, as shown in Figure 1. The impact on nanoparticle size of these co-solvents likely derives from the fact that they have similar effects as short-linker gemini surfactants when interacting with CPB in the microemulsion, giving larger particle diameter sizes [41]. It can be suggested that the three molecules act as amphiphilic moieties that insert themselves into the micelle structure. This means that, as reported by Gustafsson et al., it is probable that the CPB interhead group distance becomes shorter because of the interaction of the surfactant with these co-solvents during micelle formation. This implies that such micelles may have the following two different distances between the head groups: one dictated by the surfactant/co-solvent interaction and the other governed by the physical interactions involved in the self-assembly process [41].

**Figure 6:**
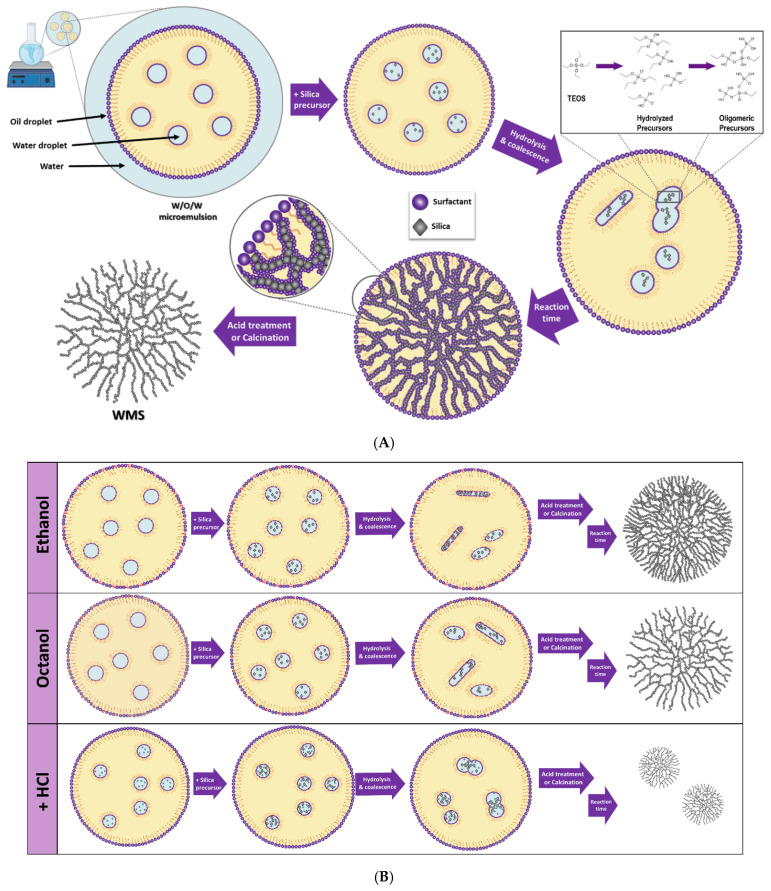
(**A**) Mechanism of formation of WMS in a bicontinuous (W/O/W) emulsion. (**B**) Effect of co-solvent and presence of HCl on micelle formation.

It can be argued that, as ethanol and glycerol have very short carbon chains, the inner water in the oil micelles (swollen reversed micelles) formed are smaller, and therefore they coalesce into narrower elongated micelles that will give rise to the pore network (Figure 6). In Figure 1, it can be appreciated that, when using ethanol as a co-solvent, the WMS showed very small pores, almost similar to those usually expected for MSN synthesized via the Stöber’s reaction. The pores of these NPs could not be accurately measured with ImageJ. This is probably because ethanol has a two-carbon chain that does not interfere much between the regular stacking of self-assembled CPB rods that will become the pores of the nanostructure. It has been previously reported for CTAB, a hydrophobic amphiphile also used as a surfactant, that at low concentrations it forms spherical micelles, while at higher concentrations, the micelles become elongated [55,56]. This is important, as it has been reported that *n*-alcohols shorter than *n*-butanol, acting as co-solvents, lead to an increase in the solubility of surfactants [57], which enhances the formation of elongated micelles and lowers the critical concentration for the transition to rod-like micelles. The latter supports the observation of smaller pores in the WMS synthesized with ethanol as a co-solvent. The NPs formed when glycerol is used are slightly smaller and start to show radial wrinkle behavior; the measured pores in the SEM micrographs are between 3 and 10 nm. The three hydroxyl groups may form hydrogen bonds between the glycerol molecules and water, which makes the incorporation into the W/O/W micelle interphase more difficult, which explains the slightly smaller diameter of these NPs compared to those synthesized in the presence of ethanol. On the other hand, glycerol is bulkier than ethanol, and probably disrupts the packing between the coalescing self-assembled CPB rods, resulting in larger pores and slight radial wrinkle behavior. When octanol was used as a co-solvent, the average size of the WMS was similar to that observed when using glycerol, but showing a more pronounced radial wrinkle structure, and pore sizes in the range between 20 and 100 nm. This observation is in agreement with the hypothesis that if the co-solvent has a longer carbon chain, such as octanol, it can interfere in the regular stacking of self-assembled CPB rods, inducing a more open wrinkled structure in the final product. The long carbon chain impedes close interaction between the elongated micelles, inducing the formation of a radial wrinkle structure and large pores. The aforementioned mechanism for WMS formation is similar to that previously proposed by other groups [41,58,59]. There are also several previous studies reporting that longer-chained *n*-alcohols (such as *n*-octanol and *n*-hexanol) act more as co-surfactants than co-solvents, because they are incorporated into the micelle’s structure [60,61,62], assembling at the interface between the micellar shell and core, strongly influencing the formation of larger micelles [63].

The use of ethylene glycol as a co-solvent yielded NPs with a size and morphology similar to those observed when using isopropanol (Figure 1). In these cases, the particle size derived from the synthesis is smaller, which probably means that these two co-solvents do not affect the water-in-oil-in-water (W/O/W) micelle formation. As ethylene glycol would be more soluble in the aqueous phase, while isopropanol would be more soluble in the hydrophobic phase, these co-solvents would not insert themselves into the micelle interphase, but instead could be acting as spacers between the inner water-in-oil micelles, allowing the formation of bigger pores and the expected formation of radial wrinkles. In addition, both alcohols could slow down the rate of silica hydrolysis compared to other *n*-alcohols, giving more time for the CPB rod-like structures to fully form at their optimal size. In the case of isopropanol, crowding around the secondary hydroxyl group could decrease the hydrolysis rates, while in the case of ethylene glycol, the same effect could be achieved by hydrogen bonding between the molecules.

Varying the amount of HCl added to the reaction mixture (Figure 2) narrows the range of sizes in this series. In both the reactions with isopropanol/urea and ethylene glycol/urea, the WMS sizes decreased when higher concentrations of acid were added. In the isopropanol/urea series, the sizes ranged between 95 and 36 nm, but as the size decreased, so did the size homogeneity, and some NPs started to lose their typical spherical morphology. From the DLS analysis, it was found that smaller WMS were more prone to aggregate in comparison to larger WMS particles. This problem was not detected for the systems using ethylene glycol/urea, which showed a size range between 98 and 59 nm, and presented a more consistent size homogeneity and spherical morphology. The observation that, with increasing concentrations of acid, the particle diameters decreased can be explained by the acceleration of the silanization reaction, by the increasing acid concentrations, which affect the coalescence of the inner water-in-oil micelles into individual particles, due to the decreasing availability of raw material for the reaction, making the products smaller. In the case of the isopropanol/urea reaction at higher HCl concentrations, the homogeneity and sphericity of the products are lost due to water-in-oil micelles coalescing unevenly and too fast. In the case of the reaction with isopropanol/HMT, it can be speculated that HMT reacts with the acid, neutralizing it and acting as a buffer, avoiding a significant pH change derived from the addition of acid, and resulting in particle sizes that are very similar in all cases and much larger than the 100 nm desired threshold.

## 4. Materials and Methods

### 4.1. Materials

Cetylpyridinium bromide (CPB, 97%), urea, cyclohexane, isopropanol, octanol, glycerol, tetraethyl orthosilicate (TEOS, 99%), concentrated hydrochloric acid (HCl, 37%), ethylene glycol, absolute ethanol, hexamethylenetetramine (HMT), and sodium hydroxide (NaOH) were purchased from Sigma-Aldrich and were used as received. Water was doubly deionized in a Milli-Q^®^ Reference ultrapure purification system, rendering conductivity in the range of 16–18 MΩ.

### 4.2. Synthesis of WMS

The synthesis of WMS was carried out according to a modified version of Moon´s methodology [42]. In short, the base was dissolved in 30 mL H_2_O_dist_ in a 500 mL round-bottom flask. The mixture was in constant vigorous agitation using a magnetic stirrer; the co-solvent, HCl, and 1 g of CPB were added, followed by 30 mL of cyclohexane. The precise quantities for each synthesis are given in Table 2 and Table 3. The mixture was allowed to mix for 30 min, after which 2.52 mL of TEOS was added dropwise within 5 min. The system was refluxed for 10 h at 90 °C. The product was then cooled, centrifuged (5000 rpm, 15 min), and washed three times with acetone and three times with water. The white product was re-dispersed in 50 mL of ethanol and 4 mL of 12 M HCl was added and refluxed at 90 °C for 24 h. The product was cooled again, centrifuged, and washed three times with acetone and three times with water. Finally, it was dried in a vacuum oven at 60 °C for 8 h, recovering a white powder at the end of the process.

### 4.3. Characterization

The characterization of the crystalline phases of the WMS nanostructures was performed by X-ray diffraction (XRD) on powder samples with a Bruker-AXS D5000 X-ray diffractometer (Cu Kα radiation source, λ = 1.5418 Å) (Karlsruhe, Germany). Measurements were collected in the 2θ angle range between 10 and 80° with a sweeping step of 0.02° and 10 s/step. The morphology was visualized with a field emission high-resolution scanning electron microscope (HR-FESEM, Tescan MAIA 3, Brno-Kohoutovice/Czech Republic), operating at 10 keV, and equipped with a Bruker XFlash 6|30 energy dispersive X-ray detector (EDS) (Berlin, Germany). Average particle size and size distribution were determined by analysis of the SEM micrographs using the ImageJ software [64]. The dispersive energy X-ray analysis (EDS) was used to determine the composition and measure the distribution of elements. Average particle size, size distribution, and zeta potential in water suspensions (1 mg/mL) were obtained using a dynamic light scattering instrument (DLS, Microtrac Nanotrac Wave II, Montgomeryville, PA, USA). Thermogravimetric analysis (TGA, Netzsch STA 2500 Regulus, Selb, Germany) under N_2_ inert atmosphere was used to determine the general thermal behavior of the materials. Fourier transform infrared (FTIR) spectra were recorded in a Carey 630 (Agilent Technologies, Santa Clara, CA, USA) spectrometer using the attenuated total reflectance (ATR) method. The BET (Brunauer–Emmett–Teller) surface area and the pore size BJH (Barett–Joyner–Halenda) were calculated by analyzing N_2_ adsorption–desorption isotherms measured at a liquid nitrogen temperature (−196 °C) using a BELSORP-mini II (MicrotracBEL, Osaka, Japan).

## 5. Conclusions

From this preliminary study, it can be concluded that the nature of the co-solvents on particle size and pore dimension affects both the particle diameter and the pore size. This is not surprising, considering that the interaction of the co-solvent with the surfactant can affect the packing at the oil–water interface, which should be important for the resulting particle morphology. Furthermore, the mechanism of formation described by different groups in recent papers [41,58,59] accurately predicts the observed phenomenon, regarding the change in particle size and homogeneity when increasing concentrations of acid are added to the reaction for the preparation of other mesoporous silica systems. Finally, WMS nanospheres with sizes less than 100 nm were successfully obtained, conserving their characteristic open-pore radial wrinkle morphology, by using ethylene glycol as a co-solvent in the presence of a given concentration of acid in the reaction mixture. To our knowledge, the use of this co-solvent has never been reported for the preparation of WMS. Further work on the evaluation of the performance of these systems as nanostructured drug delivery systems is currently under preparation.

## Figures and Tables

**Table 1:** Superficial area and porous properties of WMS systems synthesized with different HCl concentrations and varying co-solvents and bases.

	Size (nm)	Sup Area [m^2^/g] ^&^	Pore Size (nm) ^#^	Pore Size (nm) ^$^	Alcohol	Base	HCl [mM]
1	36.90 ± 3.31	302.6	3.5 * (15, 25–35) **	7.32 ± 3.25	isopropanol	Urea	1
2	43.03 ± 4.75	368.6	4–8 * (15–40) **	4.83 ± 1.65	isopropanol	Urea	0.75
3	61.94 ± 6.26	498.0	2–6 * (25–47) **	8.54 ± 1.58	isopropanol	Urea	0.5
4	95.42 ± 8.44	--	--	9.49 ± 3.16	isopropanol	Urea	0.25
5	227.27 ± 14.99	359.1	1–15 *	5.62 ± 1.58	isopropanol	HMT	1
6	237.18 ± 11.79	--	--	5.66 ± 1.58	isopropanol	HMT	0.75
7	246.64 ± 50.10	--	--	5.21 ± 0.82	isopropanol	HMT	0.5
8	271.86 ± 22.46	915.8	3.5 *	6.36 ± 1.63	isopropanol	HMT	0.25
9	59.90 ± 9.11	349.7	3–6 * (22–38) **	5.07 ± 1.21	ethylene glycol	Urea	1
10	69.15 ± 6.89	287.4	3–7 * (22–48) **	7.71 ± 3.28	ethylene glycol	Urea	0.75
11	79.94 ± 6.09	518.3	2–7 * (15–35) **	7.35 ± 2.27	ethylene glycol	Urea	0.5
12	98.26 ± 8.58	441.5	4–7 * (22–44) **	4.52 ± 1.41	ethylene glycol	Urea	0.25

^&^ Applying the BET (Brunauer–Emmett–Teller) equation. ^#^ Through the BJH (Barett–Joyner–Halenda) method. ^$^ Pore size estimated from SEM micrographs. * Main pore size distribution. ** Minor secondary pore populations.

**Table 2:** WMS synthesis specifications for different co-solvents.

Synthesis	Base	Base (g)	Co-Solvent	Co-Solvent (mL)
1	urea	0.6	isopropanol	0.920
2	urea	0.6	octanol	0.953
3	urea	0.6	ethanol	0.350
4	urea	0.6	ethylene glycol	0.335
5	urea	0.6	glycerol	0.438

**Table 3:** WMS synthesis specifications with varying HCl.

Synthesis	Base	Base (g)	Co-Solvent	Co-Solvent (mL)	HCl (mM)
1	urea	0.6	isopropanol	0.920	1.00
2	urea	0.6	isopropanol	0.920	0.75
3	urea	0.6	isopropanol	0.920	0.50
4	urea	0.6	isopropanol	0.920	0.25
5	HMT	0.14	isopropanol	0.920	1.00
6	HMT	0.14	isopropanol	0.920	0.75
7	HMT	0.14	isopropanol	0.920	0.50
8	HMT	0.14	isopropanol	0.920	0.25
9	urea	0.6	ethylene glycol	0.335	1.00
10	urea	0.6	ethylene glycol	0.335	0.75
11	urea	0.6	ethylene glycol	0.335	0.50
12	urea	0.6	ethylene glycol	0.335	0.25

## Supplementary Materials

The following are available online, Figure S1: Thermal analysis by TGA in inert conditions of Cetylpyridinium bromide, Figure S2: Pore size distribution graphs for different synthesis performed using varying co-solvents and bases in the presence of distinct concentrations of HCl, Figure S3: SEM micrographs of WMS (A) before and (B) after thermal treatment at 800 °C.

## Sample Availability

Samples of the compounds are available from the authors.
