# Supplementary material for: Effects of Co-Solvent Nature and Acid Concentration in the Size and Morphology of Wrinkled Mesoporous Silica Nanoparticles for Drug Delivery Applications
Source: Molecules. 2021 Jul 9;26(14):4186. doi: 10.3390/molecules26144186 (PMC8304942; doi:10.3390/molecules26144186)
Supplement: Supplementary file 1 [file molecules-26-04186-s001.zip › molecules-1277514-supplementary.pdf]

Effects of co-solvent nature and acid concentration in the size and morphology of wrinkled mesoporous silica nanoparticles for drug delivery applications

Jessica Andrea Flood-Garibay[1] and Miguel A. Méndez-Rojas [1,*]

[1] Department of Chemical & Biological Sciences and Laboratory of Nanotechnology and Molecular Biomedicine Research, School of Sciences, Universidad de las Américas Puebla, San Andrés Cholula, 72810 Puebla, México; jessicaa.floodgy@udlap.mx
* Correspondence: miguela.mendez@udlap.mx

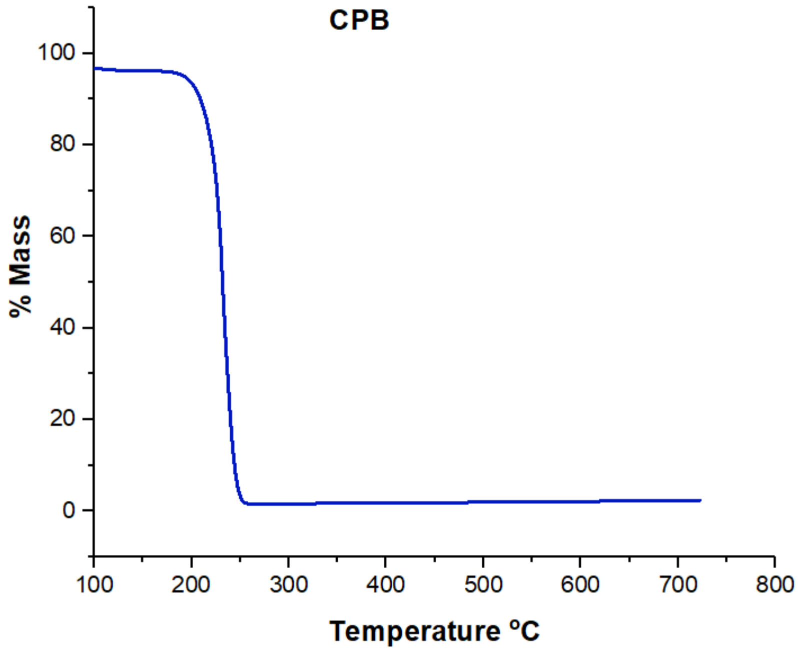

Figure S1. Thermal analysis by TGA in inert conditions of Cetylpyridinium bromide (CPB, 97%).

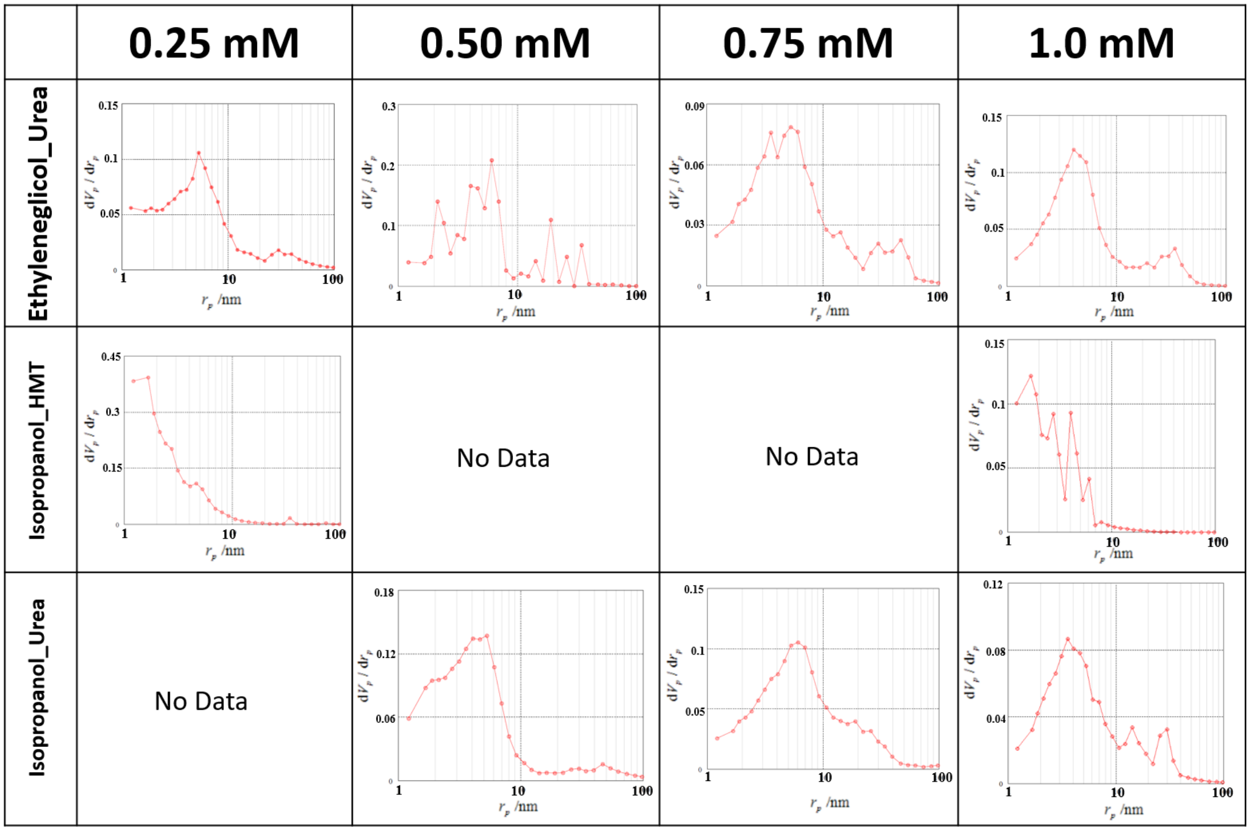

Figure S2. Pore size distribution graphs for different synthesis performed using varying co-solvents and bases in the presence of distinct concentrations of HCl.

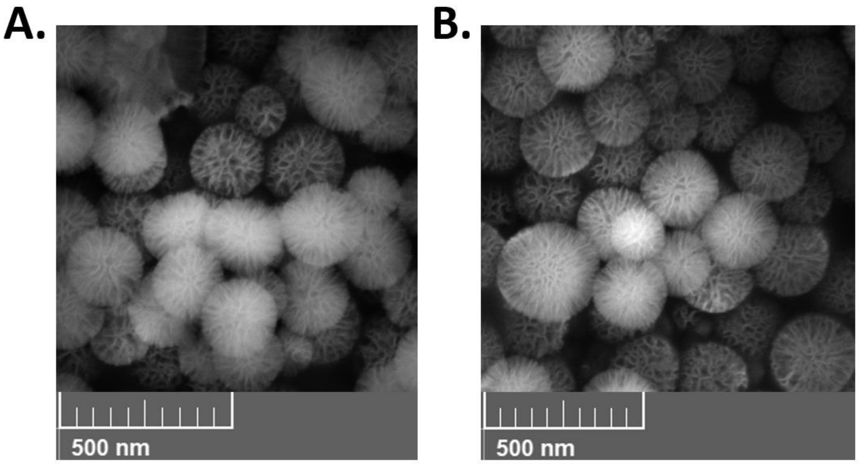

Figure S3. SEM micrographs of WMS **A.** before and **B.** after thermal treatment at 800 ^{o}C
